# Eco-conscious TLC assay for dual psoriasis drugs in plasma: a green analytical approach

**DOI:** 10.1186/s13065-025-01531-0

**Published:** 2025-06-13

**Authors:** Maimana A. Magdy, Basma H. Anwar, Nehal F. Farid, Nessreen S. Abdelhamid

**Affiliations:** Pharmaceutical Analytical Chemistry Department, Faculty of Pharmacy, Beni-Suef University, Alshaheed Shehata Ahmad Hegazy St., Beni-Suef, 62514 Egypt

**Keywords:** Green chemistry, Pentoxifylline, Spiked human plasma, Sulfasalazine, Thin layer chromatography

## Abstract

Psoriasis is one of the dermatological autoimmune diseases that involve cracking, redness, bleeding and inflammation on the surface of the skin. Sulfasalazine (SUL) is an immunity suppressing and anti-phlogistic drug. Pentoxifylline (PTN) is an immunosuppressant and vasodilator. So, the two drugs are co-administered together in the treatment protocol for psoriasis. No chromatographic analytical method was developed in the literature for the quantitative determination of SUL and PTN in their binary mixture and spiked human plasma. So, this work’s goal is to establish an environmentally friendly and selective TLC method for quantitative assay of sulfasalazine and pentoxifylline in their common mixture and spiked human plasma samples. The separation was successfully obtained using a developing system consisting of ethanol: ethyl acetate (7: 3, v/v) and 270 nm as UV scanning wavelength. Paracetamol was chosen as an internal standard to correct sampling minute variations. The obtained retardation factor values were 0.02, 0.43, 0.68 and 0.8 for plasma, pentoxifylline, sulfasalazine and paracetamol, in the same order. The resulting LLOQ for SUL and PTN were 0.3 and 0.2 µg/band, respectively. Three environmental friendliness assessment tools including analytical greenness metric approach (AGREE), eco-scale assessments, and green analytical procedure index (GAPI) were applied to evaluate the environmental safety characters of the suggested method. Validation parameters were within the accepted ranges when checked according to US-FDA guidelines to for bioanalytical method validation.

## Introduction

**Fig. 1:**
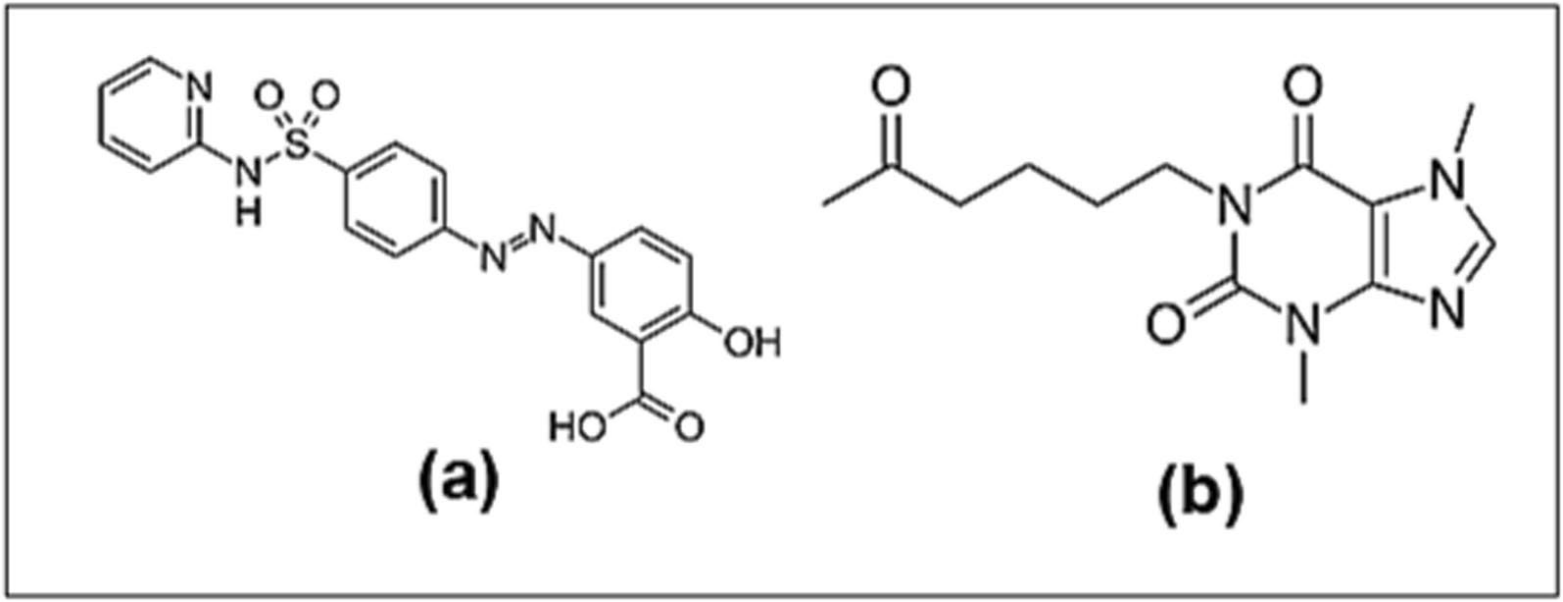
The chemical structures of **a** sulfasalazine and **b** pentoxifylline

Pentoxifylline (PTN); as presented in Fig. 1, is a blood vessel dilating agent [1]. It performs its action by enhancing the blood stream and increasing the oxygen supply to the body organs [2]. Moreover, it acts as immunosuppressant by preventing neutrophil initiation [2]. In the literature, PTN was quantitated singly or in the existence of its impurities and different drugs in different sample types using many assay methods. For example; spectrophotometry [3], colorimetry [4], HPLC [5–8] and TLC [9, 10].

Sulfasalazine (SUL); as presented in Fig. 1, is an immunity suppressing and anti-phlogistic drug. It acts by blocking T-cell and B-cell activation and synthesis [11]. In the literature, SUL was analyzed and quantitated alone or in presence of its impurities and other drugs in various matrices using many assay methods. For example; spectrophotometry [12], spectrofluorimetry [13], colorimetry [14], HPLC [15–19] and HPTLC [19, 20].

PTN and SUL are simultaneously administered for the treatment of psoriasis [21], which is defined as an autoimmune disease that involves cracking, redness, bleeding and inflammation on the surface of the skin [22]. Additionally, PTN and SUL are simultaneously administered for the treatment of pemphigus vulgaris [23, 24], which is defined as an autoimmune disease which damages skin and mucous tissues causing painful lesions and blisters [25]. Both drugs help in the treatment of psoriasis and pemphigus vulgaris through inhibition of tumor necrosis factor-alpha (TNF) synthesis [21, 23, 24].

In the literature, only principle component regression (PCR) and partial least squares regression (PLS) chemometric assays were developed for the analysis of the two drugs [26], while no chromatographic methods were developed. Thus, this research introduces a US-FDA validated TLC method for separation and quantitative assay of PTN and SUL in their common laboratory mixture and spiked human plasma samples with high selectivity and accuracy. Furthermore, the environmental friendliness of the suggested method was evaluated using three assessment methods; the analytical eco-scale assessment (ESA) [27], green analytical procedure index (GAPI) [28], and analytical greenness metric approach (AGREE) [29]. The established TLC method has the advantages over the reported chemometric method [26] of being environmentally friendly, cheaper, more selective, more sensitive and capable for quantitative assay of SUL and PTN in their common mixture and spiked human plasma samples.

## Experimental

### Instruments

- TLC plates made of aluminum with dimensions of (20 * 20 cm) coated with silica gel 60 F_254_ with thickness of 0.25 mm and particle size of 5 µm (Merck, Germany).
- Applications were done by 100 µL syringe using Camag Linomat IV applicator.
- Scanning was done using TLC scanner, model 3 S/N Camag (Muttenz, Switzerland) connected and controlled with winCATS software (version 3.15).
- Low-speed Electric Centrifuge (4000 rpm) was used to precipitate and separate the plasma protein (Zjmzym, China).
- A Scilogex MX-S vortex agitator was used (Bedfordshire, UK).

### Material and reagents

#### Pure samples

- Pentoxifylline (PTN) was granted by Alexandria Company for pharmaceuticals and chemicals, Egypt. The company certified its purity to be 99.92%.
- Sulfasalazine (SUL) was granted by Alkahira Company for Pharmaceuticals and Chemicals, Egypt. The company certified its purity to be 99.89%.
- The internal standard paracetamol (PAR) was bought from sigma Aldrich, Egypt. The company certified its purity to be 99.95%.
- Human plasma samples were funded by El-Safa laboratory (Fayoum, Egypt). The plasma samples were drawn from six healthy volunteers, between 35 and 50 years old, with normal kidney and liver functions.

#### Chemicals and reagents

- Methanol and ethanol HPLC purity grade was bought from Fischer, UK.
- Ethyl acetate was bought from EL-Nasr pharmaceutical, Chemical Co. (Cairo, Egypt).

### Standard solutions

Standard solutions with concentrations of 1 mg/mL of PTN, SUL and PAR were separately prepared using methanol as solvent.

### Blank plasma sample

One mL of human plasma was accurately introduced to 10-mL flask, and adjusted to volume with methanol solvent. The protein content of plasma was precipitated and the solution was centrifuged. Finally, the clear phase was taken.

## Procedures

### Chromatographic conditions

Camag Linomat IV applicator was used to apply ten µL samples at 10 mm from the bottom of the TLC plates as bands 3 mm in width with 6 mm distance in between. The developing system consisted of ethanol / ethyl acetate mixture (7: 3, v/v) was transferred to the developing container and left for 15 min for saturation. The plates were placed with their bottom edge immersed in the solvent mixture, covered well, and left for complete development. Finally the spots were detected at 270 nm by UV scanner.

### Establishment of calibration curves and analysis of QC samples

For pure samples: From the respective standard solution (1 mg/mL) of each drug, serial volumes equivalent to 0.2 – 1.4 mg of PTN, and 0.2 – 1.2 mg for SUL were transferred into two distinct groups of 10-mL volumetric flasks, 1 mL internal standard (PAR) standard solution (1 mg/mL) was introduced to each flask, prior to adjusting the volumes to the mark with methanol. Ten µL triplicated injections of each sample were used, followed by the same instructions mentioned under (Sect. "Chromatographic conditions"). Finally, the integrated peak area ratios (peak area of the analyzed component divided by the peak area of internal standard) were plotted against the concentrations (µg/band) to create each drug’s calibration curve.

For spiked human plasma samples: From the respective standard solution (1 mg/mL) of each drug, serial volumes equivalent to 0.2 – 1.4 mg of PTN, and 0.3 – 1.2 mg for SUL were transferred into two distinct groups of 10-mL volumetric flasks. One mL of plasma and 1 mL internal standard (PAR) standard solution were added to all of the flasks, vortexed for 1 min, prior to adjusting the volumes to the mark with methanol. The precipitated plasma protein was removed by centrifugation at 4000 rpm for 5 min. Triplicated injections each of 10 µL of the clear phase were applied on TLC plates, and the instructions mentioned under (Sect. "Chromatographic conditions") were carried out. The calibration graphs of the spiked human plasma samples were established between the integrated peak area ratios (peak area of the analyzed component divided by the peak area of internal standard) and the concentrations (µg/ band). The FDA Quality control (QC) samples including lower limit of quantitation (LLOQ), low QC (LQC), the middle QC (MQC) and the high QC (HQC) [30] were prepared and analyzed similarly for each component.

## Results and discussion

Due to its high selectivity, accuracy, and minimal sample preparation requirements, the TLC technique has become high frequently chosen for the resolution and simultaneous quantification of drug mixtures in a variety of sample natures, including dosage forms, water, soil, and biological samples like plasma, urine, and saliva [31–33]. Nowadays, the green chemistry is considered by many analytical chemists to keep the environment save and protect health [34]. Therefore, a green evaluated TLC method is introduced in this study for the simultaneous measurement of PTN and SUL in their two component mixtures and spiked human plasma samples. As a result, our approach offers a TLC method that has the advantages of being simple, green, and affordable. It also enables the detection and quantitation of the two medicines in spiked human plasma.

### Method optimization

To get the best resolution and sensitivity, a variety of parameters were investigated, including:

#### The mobile system

**Fig. 2:**
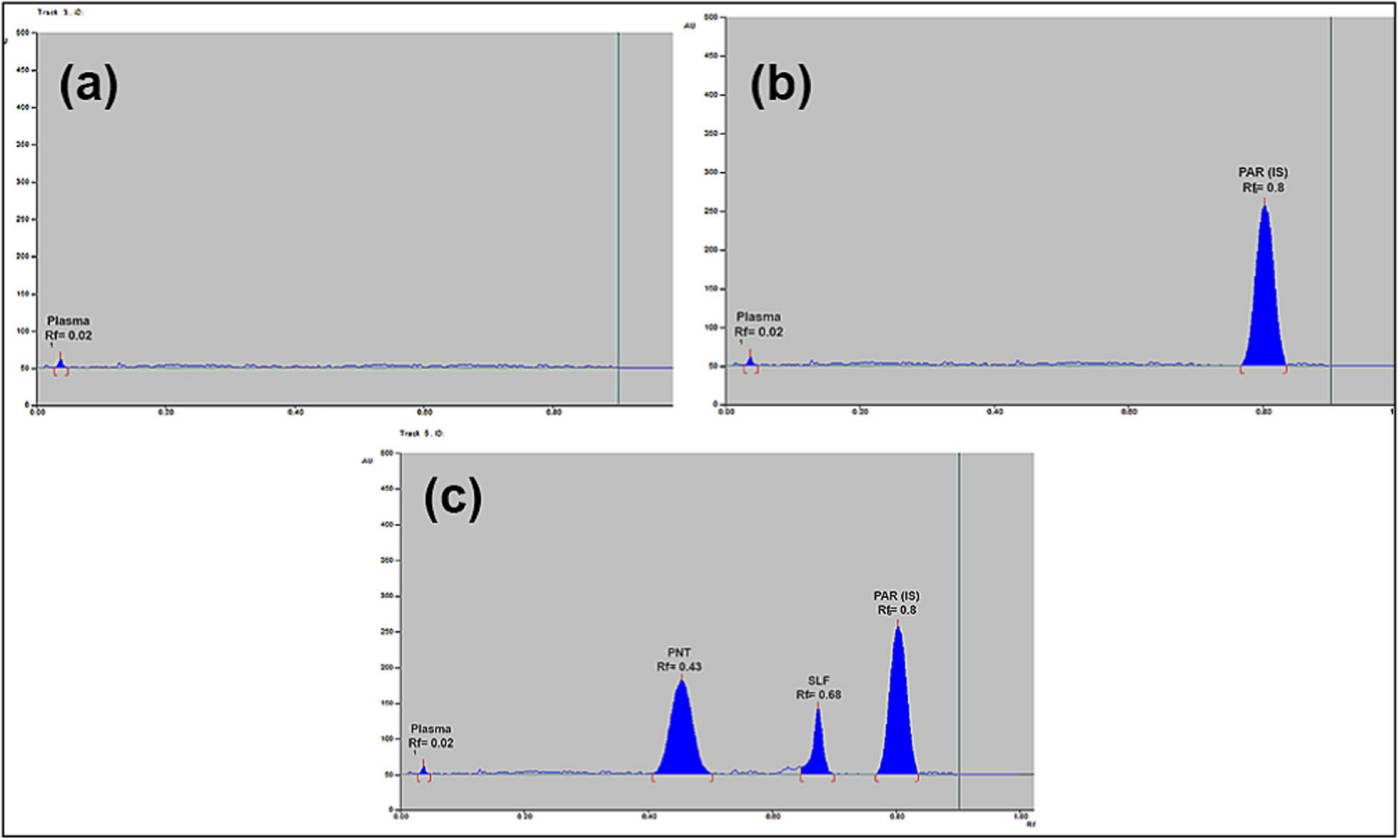
2D chromatogram of **a** blank human plasma, **b** plasma and 1 µg/band of the internal standard paracetamol, **c** spiked human plasma with 0.4 µg/band of pentoxifylline, 0.3 µg/band sulfasalazine and 1 µg/band of the internal standard paracetamol, using ethanol—ethyl acetate (7: 3, v/v) as a developing system at 270 nm

Various developing systems were tested to resolve the two components; SUL and PTN in spiked human plasma to target the most suitable resolution and selectivity, starting with green solvents such as ethanol and ethyl acetate. The first tried mixture was ethanol/ethyl acetate (1: 9, v/v) where the peaks of two drugs were separated enough from each other, but SUL was retained to the bottom line and very near to the plasma peak. Then the ratio was changed to (5: 5, v/v), where SUL moved upwards to the middle of the plate, but it was unresolved from PTN peak. After that, the ratio was changed to (7: 3, v/v), where the two drugs were completely resolved from each other and from the plasma peak. The peaks of the; PTN and SUL were symmetrical and sharp. Finally the chosen mobile system was ethanol: ethyl acetate (7: 3, v/v), as shown in Fig. 2.

#### UV scanning wavelength

**Fig. 3:**
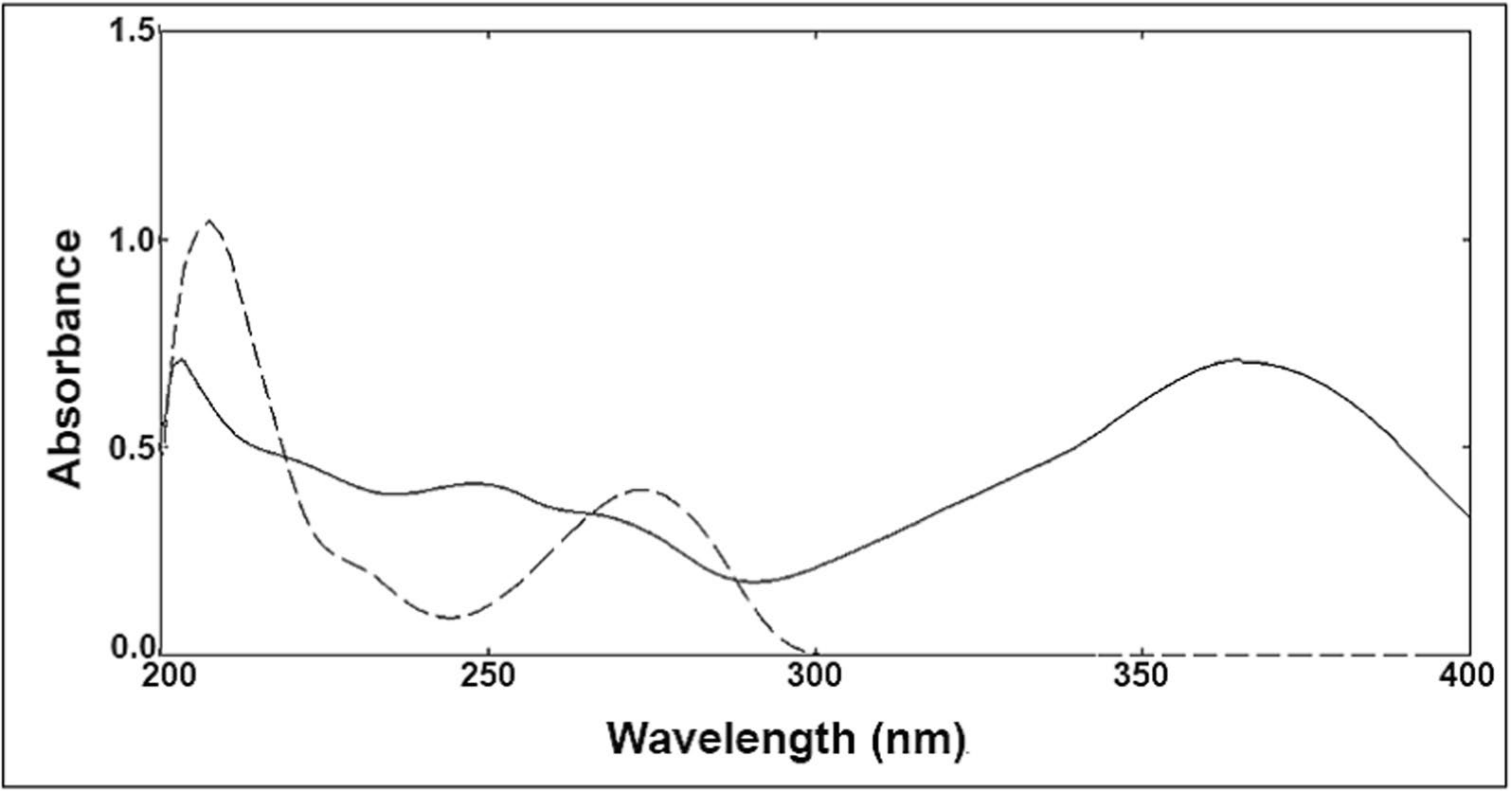
Zero order absorption spectra of 10 µg/mL of each of sulfasalazine (solid line) and pentoxifylline (dashed line) using methanol as solvent

According to UV–Vis absorption spectra of both PTN and SUL (Fig. 3), the optimal sensitivity of the suggested medications was achieved using the scanning wavelength of 270 nm (Fig. 2), after testing various wavelengths for scanning such as 245, 250, 270, and 300 nm.

#### Selection of internal standard

Different internal standards were tried; including duloxetine hydrochloride, tadalafil, dapoxetine hydrochloride, perphenazine, paracetamol and nortriptyline. The most suitable internal standard was PAR.

### Method development

The calibration graphs of the analyzed drugs were created by plotting the peak area ratio (peak area of the drug divided by peak area of IS) against the concentrations (µg/band). Linear regressions occurred in the concentration ranges of 0.2–1.4 µg/band for PTN and 0.2–1.2 µg/band for SUL. The criteria for regression equations were listed in Table 1. The obtained R_f_ values were: 0.02, 0.43, 0.68 and 0.80 for plasma, PTN, SUL and PAR, respectively, as demonstrated in Fig. 2c.

**Table 1:** Assay and method validation parameters for the determination of pentoxifylline and sulfasalazine by the proposed TLC method

Parameters	Pure		Spiked human plasma	
	PTN	SUL	PTN	SUL
Calibration range (µg/band)	0.2–1.4	0.2–1.2	0.2–1.4	0.3–1.2
Slope	0.8630	0.8286	1.1732	0.5536
Intercept	0.1849	0.0417	0.1765	0.2458
Correlation coefficient	0.9997	0.9998	0.9996	0.9997
Accuracy	98.74 ± 1.473	98.46 ± 0.813		
Robustness parameters (RSD %)^a^ - Ethanol (7 ± 1% mL) - Ethyl acetate (3 ± 1% mL) - Detection wavelength (270 ± 1 nm) - Saturation time (15 ± 5 min)	–		1.189 1.176 1.613 0.845	1.127 1.162 1.256 1.062
LLOQ (µg/band)			0.2	0.3
ULOQ (µg/band)			1.4	1.2

^a^the %RSD was calculates for the R_f_ values

### Method validation

The presented bioanalytical TLC method was validated in accordance with FDA requirements [30].

#### Calibration curves, lower and upper limits of quantification (LLOQ & ULOQ):

In the concentration ranges of 0.2–1.4 µg/band for PTN and 0.3–1.2 µg/band for SUL, linear calibration curves of plasma samples spiked with the investigated medications were obtained. As demonstrated in Table 1, LLOQ and upper ULOQ were 0.2 and 1.4 µg/ band for PTN and 0.3 and 1.2 µg/ band for SUL.

#### Accuracy, precision and quality control samples (QCs)

LLOQ, LQC, MQC, and HQC were four QC samples, validated in order to prove the accuracy and precision of the established procedure. The chosen QC concentrations were (0.2, 0.6, 0.8, and 1.0) µg/band for PTN, and (0.3, 0.6, 0.8, and 1.0) µg/band for SUL. The concentrations of the medications under study were determined using the corresponding regression equations mentioned in Table 1, and the results are displayed in Table 2. The corresponding calculated regression equations were found to be:

$${\text{PA}}_{{{\text{PTN}}}} = { 1}.{\text{1732 C}}_{{{\text{PTN}}}} + \, 0.{1765}, {\text{r}}_{{1}} = \, 0.{\text{9996 for PTN}}.$$

$${\text{PA}}_{{{\text{SUL}}}} = \, 0.{\text{5536 C}}_{{{\text{SUL}}}} + \, 0.{2458}, {\text{r}}_{{2}} = \, 0.{\text{9997 for SUL}}.$$

where PA is the integrated peak area ratio (peak area of the analyte/ peak area of IS), C is the concentrations in µg/band and r are the correlation coefficients.

**Table 2:** Intra and inter assay precision and accuracy of LLOQ, LQC, MQC and HQC of pentoxifylline and sulfasalazine in spiked human plasma samples

Component	Concentration (µg/band)^a^		Intra-day			Inter-day		
			Recovery %	RSD %	Bias %^b^	Recovery %	RSD %	Bias %^b^
PTN	LLOQ	0.2	111.97	4.372	11.97	117.63	3.147	17.63
	LQC	0.6	107.18	4.467	7.18	109.26	4.078	9.26
	MQC	0.8	108.71	2.201	8.70	108.34	2.812	8.34
	HQC	1.0	91.83	4.341	− 8.18	90.62	2.584	− 9.38
SUL	LLOQ	0.3	110.22	6.955	10.22	105.30	20.237	5.30
	LQC	0.6	92.81	4.345	− 7.19	91.58	5.648	− 8.42
	MQC	0.8	97.16	7.765	− 2.84	92.56	6.164	− 7.44
	HQC	1.0	101.60	10.890	1.60	98.29	13.946	− 1.71

^a^Average of 3 experiments

^b^Bias = [(measured concentration—nominal concentration)/nominal concentration] × 100

#### Specificity and selectivity

The TLC chromatograms in Fig. 2 demonstrated the selectivity of the new approach by demonstrating the good separation between the plasma, PTN, SUL, and internal standard PAR.

#### The freeze–thaw and Bench top stability

The stability of the studied medications was demonstrated by three freeze–thaw cycles and a bench top stability method, as indicated in Table 3.

**Table 3:** Stability results of pentoxifylline and sulfasalazine in spiked human plasma at different conditions using the proposed TLC method

The analyte	Recovery %^a^		
	Concentration (µg/band)	Three freeze thaw cycles^b^	Bench top stability
PTN	0.6	93.60	88.48
	0.8	91.43	86.29
	1.0	88.73	99.10
Mean ± % RSD		91.25 ± 2.440	91.29 ± 6.852
SUL	0.6	93.87	95.02
	0.8	95.41	87.41
	1.0	89.19	106.02
Mean ± % RSD		92.83 ± 3.239	96.15 ± 9.356

^a^Average of 3 determinations

^b^Freezing was done at -20 ºC

#### The extraction recovery

The extraction recovery was calculated and evaluated by comparing the analytical responses of the extracted samples at LQC, MQC, and HQC concentrations versus extracts of blanks spiked with the analyte post extraction (at least 3 replicates). Then the extraction recovery was calculated using this equation (extraction recovery = response of extracted / response of post extracted)*100 [30]. The accuracy of the extraction technique was proven by the values in Table 4.

**Table 4:** The extraction recovery results of pentoxifylline and sulfasalazine in spiked human plasma

PTN		SUL	
Concentration (μg/band)	% Recovery^a^	Concentration (μg/band)	% Recovery^a^
0.6	95.91	0.60	106.77
0.8	100.44	0.80	104.51
1.0	90.28	1.00	105.96
Mean ± % RSD	95.54 ± 5.090	105.75 ± 1.145	

^a^Average of 3 determinations

#### System suitability parameters

By computing various metrics, including resolution, the tail of the peaks, and selectivity factor (α), the system's suitability was assessed. The results were evaluated in accordance with the authorized ranges [35], as shown in Table 5.

**Table 5:** Parameters of system suitability of the developed TLC method for the determination of pentoxifylline and sulfasalazine

Parameters	Plasma	PTN		SUL		PAR	Reference value [35]
Capacity factor (K')	–	1.00		1.10		1.00	0–10
Symmetry factor	–	1.325		0.471		0.250	~ 1
Resolution (Rs)	13.111		4.080		2.381		> 1.5
Selectivity (α)	36.981		2.813		1.884		> 1

#### Robustness

By making minor, deliberate modifications in many criteria, including the developing system ratios, the scanning wavelength, and the saturation duration, the robustness of the presented TLC method was examined. Table 1 presented the results.

### Greenness assessment of the developed TLC method

#### Eco-scale assessment (ESA)

The analytical eco scale scores are 90 for the suggested TLC method (Table 6), which are considered excellent scores and greatly indicate the method eco friendliness [27].

**Table 6:** Eco-scale penalty points of the developed TLC method for simultaneous determination of pentoxifylline and sulfasalazine in spiked human plasma

The reagents			
Items	Amount	Hazard^b^	Total penalty points^c^
ethanol	1 (1.05 ~ < 10 mL)	2 (1 pictograms, danger)	2
Ethyl acetate	1 (0.45 ~ < 10 mL)	4 (2 pictograms, danger)	4
The Instruments			
Energy used (UV—scanner)	1 (< 1.5 kWh per sample)		
Occupational hazard	0		
Waste^a^	3 (1.5 ~ 1–10 mL)		
Total penalty points	Σ 10		
Analytical Eco-scale score	90		

GAPI tool	AGREE tool
	

^a^Waste = the volume of mobile phase/ No. of spots per TLC plate

^b^Hazard penalty points = No. of pictograms × signal. The signal maybe warning = 1 or danger = 2

^c^The total penalty points = the amount penalty points × hazard penalty points

#### Green analytical procedure index (GAPI)

The GAPI pictogram of the suggested TLC method displays four green segments, nine yellow segments, and two red segments, as shown in Table 6. These results indicate that the method is satisfyingly green and safe to the environment [28]. Area number 1 (which stands for sample collection) took the red color because it is offline analysis, while area number 15 (which stands for waste treatment) took the red color because no waste treatment was performed, as illustrated in Table 6.

#### Analytical greenness metric approach (AGREE)

The AGREE score was 0.76 for the suggested TLC method (Table 6). The resulted green color appeared in the centers of the resulting pictograms, indicates that the method is green enough [29]. Area number 3 (which stands for the positioning of the analytical devices) took the red color because it is an offline analysis, as illustrated in Table 6.

## Conclusion

For the quantification of PTN and SUL binary combination and spiked human plasma samples, a green-sensitive TLC technique was created employing PAR as an internal standard. The resulting LLOQ for PTN and SUL were 0.2 and 0.3 µg/band, respectively. The technique was sensitive, easy to use, quick, economical, environmentally benign, and less destructive to the environment. All validation criteria complied with FDA acceptance standards. The new TLC process is environmentally safe and green, according to the results of a three greenness evaluation tools. Accordingly, the proposed method can be utilized for routine analysis in quality control laboratories for the quantitative analysis of PTN and SUL in their binary mixture and spiked human plasma with satisfied accuracy and precision.

## Data Availability

The datasets used and/or analyzed during the current study are available from the corresponding author upon reasonable request.
